# New Aspects on the Treatment of Retinopathy of Prematurity: Currently Available Therapies and Emerging Novel Therapeutics

**DOI:** 10.3390/ijms23158529

**Published:** 2022-08-01

**Authors:** Juhee Ryu

**Affiliations:** 1 Vessel-Organ Interaction Research Center, College of Pharmacy, Kyungpook National University, Daegu 41566, Korea; juheeryu@knu.ac.kr; Tel.: +82-539508583; 2 Research Institute of Pharmaceutical Sciences, College of Pharmacy, Kyungpook National University, Daegu 41566, Korea

**Keywords:** retinopathy of prematurity, pharmacotherapy, retinal neovascularization, preterm infant, retinal vascular disease

## Abstract

Retinopathy of prematurity (ROP) is a rare proliferative ocular disorder in preterm infants. Because of the advancements in neonatal care, the incidence of ROP has increased gradually. Now, ROP is one of the leading causes of blindness in children. Preterm infants with immature retinal development are exposed to supplemental oxygen inside an incubator until their cardiopulmonary system is adequately developed. Once they are returned to room air, the relatively low oxygen level stimulates various angiogenesis factors initiating retinal neovascularization. If patients with ROP are not offered adequate and timely treatment, they can experience vision loss that may ultimately lead to permanent blindness. Although laser therapy and anti-vascular endothelial growth factor agents are widely used to treat ROP, they have limitations. Thus, it is important to identify novel therapeutics with minimal adverse effects for the treatment of ROP. To date, various pharmacologic and non-pharmacologic therapies have been assessed as treatments for ROP. In this review, the major molecular factors involved in the pathogenesis of ROP, currently offered therapies, therapies under investigation, and emerging novel therapeutics of ROP are discussed.

## 1. Introduction

Retinopathy of prematurity (ROP), a rare proliferative vascular disease in the retina of preterm infants, has become one of the main causes of childhood blindness around the world. Retinal vessels start to develop at 16 weeks of gestation, beginning from the center to the peripheral retina and completing around 40 weeks of gestation [1]. Therefore, full-term infants are generally born with nearly complete retinas, whereas preterm infants are born with immature retinas [2]. The incidence of ROP is closely associated with gestational age and birth weight. Preterm infants born with low gestational age or birth weight tend to have a high incidence of ROP [3,4]. Since the number of patients with ROP has increased due to the high survival rate of preterm infants owing to advancements in neonatal care, it is necessary to identify the optimal therapy for ROP [5].

ROP can be classified using several parameters: zone, stage, extent, and plus disease. Zone indicates the location of ROP from the center of the optic disc (Zone I) to the outer crescent (Zone III). Stage denotes the severity of ROP from mild (stage 1) to most severe (stage 5). Extent refers to retinal surface divided in twelve sections, similar to the 12 h clock. The presence or absence of plus disease, defined as the dilation and tortuosity of posterior vasculature in the retina, is a key factor in determining the severity of ROP. In addition, aggressive ROP defined as quickly progressing ROP in zone I or posterior zone II was introduced in the updated classification of ROP [6]. Treatment of ROP should be initiated when patients are diagnosed as type I ROP, which includes any ROP stage with plus disease in Zone I, stage 2/3 with plus disease in Zone II, or stage 3 without plus disease in Zone I [7]. The current standard treatment of ROP is laser therapy of the peripheral avascular retina. In addition, anti-vascular endothelial growth factor (VEGF) treatments have been used as a promising treatment option [8]. However, there are very few available pharmacologic options to treat ROP and these therapies have limitations. Thus, it is critical to discover novel therapeutics for ROP. Recently, non-coding RNA therapy, cell therapy, and gene therapy have received attention from many researchers as emerging therapeutics or therapeutic strategies. Thus, in this article, we will discuss the major factors involved in the pathogenesis of ROP, currently used therapies, treatments under investigation, and potential treatments of ROP.

## 2. Major Molecular Factors Involved in Pathogenesis of Retinopathy of Prematurity

**Figure 1:**
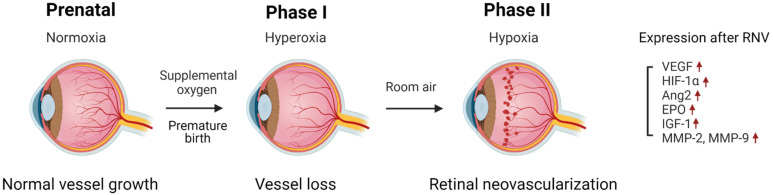
Pathogenesis of retinopathy of prematurity (ROP). Before a premature birth occurs, normal vessels develop in utero. In phase I, premature infants are exposed to relative hyperoxia due to supplemental oxygen; as a result, normal vessel growth stops and vessel loss can occur. Additionally, premature infants no longer receive nutrients and growth factors from their mothers. In phase II, infants are placed back to room air, a relative hypoxic state. As a result, retinal neovascularization (RNV) is initiated, and hypoxia inducible factor-1α (HIF-1α) vascular endothelial growth factor (VEGF) are activated. Moreover, the expressions of the genes encoding angiopoietin (Ang), erythropoietin (EPO), insulin-like growth factor (IGF), and metalloproteinase (MMP) are upregulated.

The pathogenesis of ROP develops in a two-step process, phases I and II [9]. In phase I, exogenous stress such as hyperoxia and deficiency of maternally derived factors including insulin-like growth factor-1 delays the development of normal retinal vessels in preterm infants, causing vessel loss [2,10]. Supplemental oxygen is provided to premature infants that can cause hyperoxia. As a result, VEGF expressed by astrocytes is downregulated and oxygen-induced angiogenic factors such as erythropoietin are inhibited in a high oxygen state. Suppression of these angiogenic factors can lead to shrinkage of retinal vessels and may interrupt normal vessel development in the retina [11,12,13]. Subsequently, after preterm infants are returned to ambient air, a relatively hypoxic state, angiogenesis-related factors such as VEGF and erythropoietin are upregulated in avascular regions in the retina and promote the generation of abnormal retinal vessels during phase II (Figure 1) [14,15]. Hypoxia may accelerate the degeneration of astrocytes and the expression of VEGF can be upregulated by neurons of ganglion cells [13]. As a result, retinal neovascularization (RNV) can develop in the retina and infants may experience retinal detachment and a permanent loss of vision in severe cases [16]. Although many angiogenesis-related factors are involved in RNV, we will focus on several major factors (Figure 1).

### 2.1. Vascular Endothelial Growth Factor

VEGF is an important player in retinal vessel development and ROP progression [17]. Stimulation of hypoxia-induced transcription factors can upregulate VEGF gene expression [18]. Although the VEGF family consists of various members, such as VEGF-A, -B, -C, -D, and placental growth factor (PlGF), VEGF-A is mainly involved in retinal angiogenesis [19,20]. Although there are various VEGF receptors, including VEGF-R1, -R2, and -R3, the binding of VEGF-A to VEGF-R2 primarily regulates the angiogenesis pathway [21]. In an ROP mouse model, VEGF-R2 levels are elevated during hypoxia in the avascular region of the retina, whereas VEGF-R1 levels are not significantly changed [22].

### 2.2. Hypoxia-Inducible Factor-1

Hypoxia inducible factor-1 (HIF-1), a transcriptional factor composed of two subunits such as α and β subunits, is a key factor in oxygen homeostasis. HIF-1α dimerizes with HIF-1β and initiates transcription of various angiogenic genes including VEGF. HIF-1α is downregulated during the hyperoxic state, phase I, whereas it is upregulated during the hypoxic state, phase II [23]. Whereas HIF-1α is hydroxylated by prolyl hydroxylase (PHD) and is readily degraded by proteasome in normoxic state, HIF-1α is accumulated during a hypoxic state since the activity of PHD is inhibited [24]. PHD knockout mice showed less vessel loss during hyperoxia [25], whereas suppression of HIF-1α using a HIF-1α inhibitor reduced retinal neovascularization in hypoxia [26]

### 2.3. Angiopoietin

Angiopoietins (Angs) are vascular growth factors regulating vascular remodeling. There are two types of Angs, Ang1 and Ang2, that bind with Tie2, a tyrosine kinase receptor [27]. Whereas Ang1 maintains vessel stability, Ang2 is mainly involved in vascular angiogenesis [28,29]. Vitreous level of Ang2 is upregulated in patients with late-stage ROP [30]. Moreover, Ang2 is increased by hypoxia and VEGF stimulation [31]. Furthermore, the inhibition of Tie2 and VEGF simultaneously reduces angiogenesis more than that of VEGF alone [27].

### 2.4. Erythropoietin

Erythropoietin, a glycoprotein hormone, is important in visual function development, and possesses neuroprotective properties [32]. Erythropoietin promotes angiogenesis in vitro and in vivo [11,33,34]. Additionally, a positive correlation between EPO and incidence of ROP was revealed in retrospective study of humans [35].

### 2.5. Insulin-like Growth Factor-1

Insulin-like growth factor 1 (IGF-1), a mitogenic hormone critical for fetal development, regulates various processes, including growth, angiogenesis, differentiation, and metabolism [36,37,38]. Preterm infants with low levels of IGF-1 are associated with a high incidence of ROP [10]. IGF-1 regulates vascular development in retina by modulating the mitogen-activated protein kinase and protein kinase B pathways in VEGF signaling [39,40]. Thus, IGF-1 may be important for optimal activation of VEGF signaling pathway in vascular endothelial cells. Additionally, restoring IGF-1 to the normal level in utero was found to inhibit ROP progression [41].

### 2.6. Metalloproteinase

Matrix metalloproteinases (MMPs), which degrade the extracellular matrix, are involved in angiogenesis [42,43]. Mice with an MMP-2 gene knockout showed less RNV than wild-type mice [44]. Moreover, expression of MMP-2 and MMP-9 genes were increased in the mouse retina in an in vivo ROP model. The suppression of MMPs inhibits retinal angiogenesis in vivo, whereas the upregulation of proteases, including MMPs, exacerbates ROP [45].

## 3. Current Therapies of ROP

Laser photocoagulation is the most common established therapy for ROP. Anti-VEGF drug can be administered as monotherapy or used with laser therapy (Table 1). Anti-VEGF therapy has similar efficacy to laser therapy. Moreover, anti-VEGF treatments have a lower risk for myopia and fewer adverse ocular effects than laser treatment [46,47]. Although anti-VEGF drugs are frequently used to treat ROP, they may have potential to exert long-term systemic adverse effects on other organs; thus, the recommended dosage for anti-VEGF agents is still under investigation [48,49,50].

### 3.1. Pharmacologic Treatment: Anti-VEGF Therapy

#### 3.1.1. Bevacizumab

Bevacizumab, an FDA-approved recombinant humanized antibody for colon cancer, was the first anti-VEGF agent investigated for ROP treatment [58]. Bevacizumab is more effective in patients with zone I, stage 3 ROP with plus disease than laser therapy, according to The Bevacizumab Eliminated the Angiogenic Threat of Retinopathy of Prematurity (BEAT-ROP) study. Infants administered with intravitreal bevacizumab had fewer ROP recurrences and adverse ocular outcomes, such as macular dragging than those treated with laser therapy [51]. In addition, the bevacizumab treatment group had fewer incidences of high myopia than the laser therapy group in a follow-up clinical trial of BEAT-ROP [46].

#### 3.1.2. Ranibizumab

Ranibizumab is another anti-VEGF inhibitor and a recombinant humanized monoclonal IgG1 antibody that was recently approved for the treatment of ROP for the first time in Europe and Japan [59]. Currently, ranibizumab is the FDA-approved drug for wet age-related macular degeneration and diabetic retinopathy [60,61]. Compared to bevacizumab, ranibizumab has a higher affinity for VEGF, exerts less systemic effects, and has a shorter half-life [52,53]. Meanwhile, ROP recurs more frequently with the ranibizumab treatment than bevacizumab [52]. Additionally, the efficacy of low (0.1 mg) or high-dose (0.2 mg) ranibizumab was compared with that of laser therapy in an open-label randomized controlled trial. The patients in the high-dose ranibizumab group had a higher treatment success rate and fewer ocular adverse events than those in the laser therapy group, suggesting the superiority of high-dose ranibizumab therapy to laser therapy [47].

#### 3.1.3. Pegaptanib

Pegaptanib sodium, an RNA aptamer bound to VEGF-165, has been studied for ROP treatment. Autrata et al. reported that a combination of pegaptanib and laser therapy achieved a more favorable ocular outcome in patients with stage 3+ ROP than laser therapy alone. In addition, the laser therapy group had a higher recurrence rate of ROP than the combination therapy group [54].

#### 3.1.4. Aflibercept

Aflibercept, a recently developed anti-VEGF medication, is a recombinant fusion protein targeting VEGF-A, VEGF-B, and placental growth factor [55]. Since aflibercept binds to multiple targets involved in the VEGF signaling pathway, it is considered the most potent anti-VEGF drug [62]. In addition, according to several studies, the intravitreal administration of aflibercept is effective and safe in patients with type 1 ROP [56,57]. Moreover, Sukgen and Koçluk compared the clinical outcome of aflibercept to that of ranibizumab in an observational study. They found that the patients in the ranibizumab group experienced a higher recurrence rate of ROP than those in the aflibercept group [63].

### 3.2. Non-Pharmacologic Treatment: Laser Therapy and Surgery

Conventional laser therapy is performed using diode or argon. Cryotherapy has not been commonly used to treat ROP since late 1980s because it causes more inflammation than laser therapy [64]. Additionally, laser therapy was shown to achieve more favorable ocular outcomes than cryotherapy [65,66]. As a result, laser therapy replaced cryotherapy and it has been used as a treatment for ROP longer than any anti-VEGF agents. However, laser therapy may cause a higher incidence of myopia and undesirable ocular outcomes than anti-VEGF therapy [46,47,51,67,68]. Therefore, treatments with a minimal destructive outcome are needed. When ROP aggravates to retinal detachment, ROP stage 4 or 5, surgery such as scleral buckling or vitrectomy can be performed to retain vision and reattach the retina [64].

## 4. ROP Therapies under Investigation

There are various agents that have been investigated for their effects in preclinical studies and clinical trials of ROP. Whereas some therapies have shown beneficial effects in ROP, other therapies have inconclusive reports in ROP studies (Table 2). From the ROP therapies under investigation, beta-blockers, caffeine, polyunsaturated fatty acids, and vitamin A are found to be effective in preventing ROP progression (Table 3).

### 4.1. ROP Therapies under Investigation with Partially Proven Results

#### 4.1.1. Beta-Blocker

Propranolol, a nonselective beta-adrenergic receptor blocker, has been studied for ROP in several studies. Propranolol is used to treat infantile hemangioma due to its inhibition of VEGF; therefore, it was tested for RNV [72]. The oral administration of propranolol was found to reduce ROP progression [69,70,73]; however, serious adverse effects, such as bradycardia and hypotension, were observed [70]. Meanwhile, other routes of administering propranolol, such as eye drops, were assessed for ROP. Filippi et al. found that 0.2% propranolol eye drops reduced ROP progression without causing serious adverse effects [71]. Additionally, the effect of propranolol in prevention of ROP progression was assessed in two meta-analysis [72,74]. Administration of oral propranolol in infants with ROP reduced the use of anti-VEGF drugs and laser therapy and the progression of ROP to the advanced stage [72]. Similarly, oral propranolol was reported to be effective in preventing progression of ROP to severe stage in premature infants in another meta-analysis [74]. Although propranolol has shown favorable effects in the prevention of severe ROP, it is necessary to conduct randomized controlled studies to assess the long-term outcomes and optimal dose and duration of propranolol treatment. 

#### 4.1.2. Caffeine

Caffeine, a commonly used drug for apnea of prematurity, was reported to inhibit the progression of ROP in the Caffeine for Apnea of Prematurity trial [78]. In addition, caffeine downregulates angiogenesis-related factors, such as VEGF and MMPs [75,76]. Moreover, the administration of caffeine was found to reduce ROP progression in a clinical trial and meta-analysis [77,78].

#### 4.1.3. Polyunsaturated Fatty Acids

Polyunsaturated fatty acids, including docosahexaenoic acid and arachidonic acid, are critical components of the retina and brain. Deficiency in polyunsaturated fatty acids can cause vascular complications [86,87]. Since preterm infants born before the third trimester cannot obtain polyunsaturated fatty acids from their mothers, they are at high risk for ROP [16]. Supplementation with polyunsaturated fatty acids was reported to inhibit pathological retinal neovascularization in oxygen-induced retinopathy (OIR) mice model [79]. Similarly, beneficial effects of polyunsaturated fatty acids were reported in a clinical trial and meta-analysis. Intake of polyunsaturated fatty acids improved visual acuity in infants and reduced the risk of severe ROP [80,81].

#### 4.1.4. Vitamin A

Vitamin A, a fat-soluble vitamin, is an important factor in maintaining visual functions [88]. Vitamin A reduces retinal angiogenesis in the OIR rat model of ROP by inhibiting VEGF production [84]. In addition, it was found to decrease the progression and incidence of ROP in various clinical trials and a meta-analysis [82,83,85].

### 4.2. ROP Therapies under Investigation with Contradictory Results

#### 4.2.1. Antioxidants

Antioxidants, such as vitamin E, D-penicillamine, and lutein, may not be recommended for the prevention or treatment of ROP. Since the production of reactive oxygen species may impair the mitochondrial system of the retina in preterm infants, the efficacy of antioxidant supplements has been explored [89]. According to a meta-analysis of clinical trials, vitamin E reduces the risk of ROP but increases the risk of sepsis [90]. Therefore, high-dose vitamin E is not recommended due to the risk of sepsis. D-penicillamine was also investigated for its effects on ROP; however, its significant reduction of ROP was not observed in clinical trials. Therefore, D-penicillamine is not recommended as a pharmacologic agent for preventing ROP [91]. In a meta-analysis conducted by Cota et al., lutein, a primary carotenoid in the macular pigment, was investigated for its effect on ROP and was revealed to be ineffective in preventing ROP. They reported that oral supplementation of lutein does not decrease the incidence and mortality of ROP [92].

#### 4.2.2. Corticosteroids

The use of corticosteroids in ROP remains controversial. Two studies reported that corticosteroid prevented progression of ROP to severe stage in cohort studies [93,94]. On the other hand, inhaled corticosteroid was reported to have no effect on the incidence of ROP [95]. Although the late administration (more than 7 days after birth) of postnatal corticosteroids to preterm infants increases the risk of severe ROP, the early administration (fewer than 8 days after birth) of postnatal corticosteroids is beneficial for reducing the incidence of ROP [96,97]. Meanwhile, Termote et al. found that postnatal hydrocortisone use did not increase the risk of ROP, whereas the prolonged use of hydrocortisone in ROP patients increased the risk of severe ROP [98]. Thus, future studies with various time periods of drug administration and large study populations may be necessary to verify the effect of corticosteroids.

#### 4.2.3. Light

Light exposure is thought to influence ROP; therefore, various studies have assessed the effect of light exposure on the incidence or development of ROP. In the randomized clinical trial conducted by the Light Reduction in Retinopathy of prematurity Cooperative group, light reduction was found to have no effect on the incidence of ROP [99]. Additionally, a meta-analysis, which investigated the relationship between the reduction of light intensity and occurrence of ROP using four randomized clinical trials, concluded that a decrease in light exposure within 7 days after birth did not affect the incidence of ROP [100]. Recently, the effect of red-light exposure in the prevention of ROP was investigated. Although 670 nm red-light exposure reduced ROP in vivo [101], the severity and survivability of ROP were similar between the red-light treatment group and the control group in a randomized clinical trial [102].

#### 4.2.4. Non-Steroidal Anti-Inflammatory Drugs

Ibuprofen and indomethacin, non-steroidal anti-inflammatory drugs, have been studied in an in vivo OIR model commonly used for research on ROP. However, there have been conflicting reports regarding the effect of non-steroidal anti-inflammatory drugs on ROP. For example, ibuprofen was found to be effective in reducing RNV by inhibiting VEGF signaling in an OIR rat model [103]. In contrast, Bhatt-Mehta and Schumacher reported that ibuprofen intake was not associated with the severity of the ROP in a retrospective cohort study [104]. Meanwhile, indomethacin was found to improve ROP without influencing normal retinal development in an OIR mouse model [105]. On the other hand, the administration of high-dose indomethacin was found to be associated with a higher incidence of ROP in a clinical trial than administration of low-dose indomethacin [106]. In addition, topical ketorolac has been investigated as a preventive or therapeutic treatment for ROP. Although a ketorolac ophthalmic solution was found to lower the risk of ROP progression without causing serious adverse events in a preliminary clinical trial [107], Giannantonio et al. reported that ketorolac ophthalmic drops were ineffective in preventing severe ROP [108].

#### 4.2.5. Oxygen

Modulation of oxygen levels has been tested for its effects on ROP; however, the optimal oxygen level to inhibit ROP progression remains to be determined. In the Supplemental Therapeutic Oxygen for Prethreshold Retinopathy Of Prematurity (STOP-ROP) clinical trial, the supplemental oxygen group (96–99% O_2_) exhibited a lower rate of ROP progression than the conventional group (89–94% O_2_), although the difference was statistically insignificant. However, infants in the oxygen supplementation group experienced a higher incidence of pulmonary adverse effects, such as pneumonia, chronic lung disease, and longer hospitalization than infants in the conventional group [109]. On the other hand, oxygen restriction therapy has been investigated in several studies. Compared with the liberal oxygen therapy group (91–95% O_2_), the preterm infants in the oxygen restriction therapy group (85–89% O_2_) had a similar incidence of severe ROP but had a higher risk of mortality in a meta-analysis [110].

#### 4.2.6. Serum Insulin-like Growth Factor 1 

IGF-1 is an important growth hormone provided by the mother in utero. Since preterm infants cannot produce IGF-1 on their own, they display a low level of IGF-1. Since a low level of IGF-1 is associated with a high risk of ROP [111], there have been various attempts to increase IGF-1 levels in preterm infants. The effect of breastfeeding on IGF-1 levels was assessed. Serum IGF-1 levels were found to increase in breastfed preterm infants [112,113]. In addition, human milk intake reduces the occurrence and progression of ROP regardless of the amount, according to a meta-analysis [114]. Several studies have investigated the effect of IGF-1 supplementation on the prevention or treatment of ROP. For example, a neonatal mice group administered with recombinant human insulin-like growth factor (rhIGF-1) have a lower incidence of ROP than a neonatal mice group that received a placebo [115]. In addition, the effect of rhIGF-1 and rhIGF-1-binding protein-3 (rhIGFBP-3) on the prevention of ROP was investigated in a phase II clinical trial. However, the infusion of rhIGF-1/rhIGFBP-3 did not reduce the incidence and severity of ROP [116]. Therefore, large prospective studies may be necessary to determine the effects of IGF-1.

## 5. Potential Therapeutics and Therapeutic Strategies of ROP

### 5.1. Non-Coding RNAs

**Figure 2:**
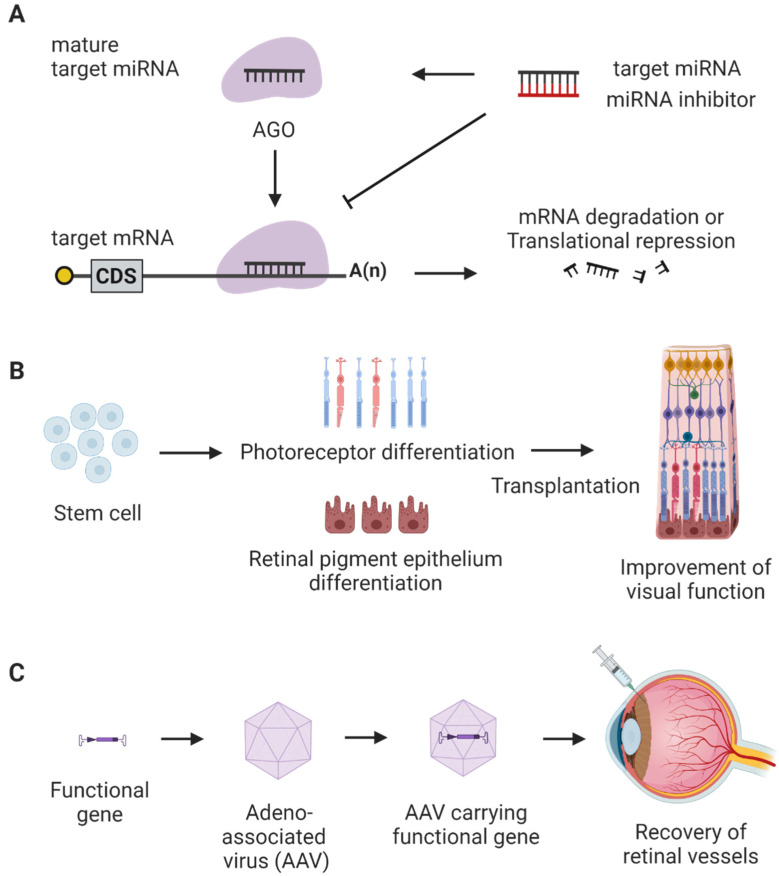
Potential therapies for retinopathy of prematurity (ROP). Examples of non-coding RNA therapy, cell therapy, or gene therapy are shown. (**A**) microRNA inhibitor can be used to suppress target microRNA. In a canonical pathway of microRNA biogenesis, single-stranded mature microRNA is loaded into ARGONAUTE (AGO) protein. Then AGO-microRNA complex binds to target messenger RNA inhibiting its action. When microRNA inhibitor is introduced, it binds to its target microRNA specifically and prevents messenger RNA degradation or translational attenuation. (**B**) Stem cells can be differentiated to photoreceptors or retinal pigment epithelium. These differentiated cells can be transplanted to the retina and visual function can be improved. (**C**) Functional gene having anti-angiogenic effects can be incorporated in an adeno-associated virus vector (AAV). AAV carrying anti-angiogenic genes may be directly administered to the retina using intravitreal injection to treat ROP.

Non-coding RNAs that are generally not involved in translation regulate diverse diseases including retinal diseases (Figure 2) [117,118,119,120]. Non-coding RNAs, including microRNAs, long non-coding RNAs, and circular RNAs, vary in length [121]. MicroRNAs, short non-coding RNAs with length around 22 nucleotides, can bind to messenger RNA and inhibit or block translation. Long non-coding RNAs with lengths longer than 200 nucleotides can regulate gene expression or modify chromatin. Circular RNAs with variable lengths often act as microRNA sponges and can indirectly affect the processing of messenger RNA [117]. 

Among the non-coding RNAs, microRNAs have been most extensively investigated in ROP. miR-410 was found to target VEGF-A, inhibiting retinal angiogenesis in the mouse OIR model. The administration of miR-410 eye drops was reported to suppress RNV [122]. In addition, Liu et al. reported that expression of miR-150 was downregulated in the mouse OIR model of ROP. miR-150 directly targets various genes related to angiogenesis, such as those encoding C-X-C chemokine receptor type 4, Delta-like ligand 4, and Frizzled-4. Overexpressing the miR-150 inhibits RNV, whereas suppressing miR-150 promotes retinal angiogenesis in vivo [123].

The role of long non-coding RNA Malat in ROP has also been studied. Suppressing long non-coding RNA Malat reduces RNV in the mouse OIR model [124]. In addition, Xia et al. has uncovered the pro-angiogenic role of long non-coding RNA Malat in vitro and in vivo. They found that long non-coding RNA Malat acted as a miR-124-3p sponge, regulating early growth response protein 1 in ROP [125]. TUG1, another long non-coding RNA, is also involved in oxygen-induced RNV. Knocking down TUG1 suppresses retinal angiogenesis by sponging miR-299-3p, subsequently reducing VEGF-A levels and RNV [126]. 

Furthermore, circZNF609 is the first circular RNA studied in the ROP model. The expression of circZNF609 is increased after hypoxia induction. Knockdown of circZNF609 inhibits the progression of retinal angiogenesis and vessel loss in vivo. This study revealed that circZNF609/miR-615-5p/myocyte-specific enhancer factor 2A regulated endothelial cells [127]. Recently, circPDE4B, another circular RNA involved in ROP, has been reported. Expression of circPDE4B was downregulated in the ROP model. circPDE4B was found to interact with miR-181c and subsequently regulate von Hippel-Lindau and VEGF-A [128].

Although ROP can be treated with laser photocoagulation, novel therapeutic approaches such as non-coding RNAs may provide less destructive treatment options to treat ROP. Non-coding RNA delivery via intravitreal injection, a commonly used method to treat retinal diseases, may be safer treatment to treat ROP than laser therapy [129]. Additionally, topical eye drops, relatively noninvasive treatments, can be employed to deliver non-coding RNAs [130]. 

Targeting non-coding RNAs have received attention over the past decade and various non-coding RNA-related therapeutics have been on clinical trials. From various types of non-coding RNAs, microRNA-targeting drugs have been extensively investigated. There are several microRNA mimics and antimiRs that have been studied in clinical trials. For example, the effect of MRX34, the miR-34 mimic, was investigated in the phase I clinical trial of advanced solid cancer [131]. Moreover, the safety and activity of MesomiR-1, the miR-16 mimic, was tested in phase I clinical trials of malignant pleural mesothelioma [132]. In addition, the effect and safety of RG-101 and miravirsen, miR-122 antimiRs, was studied in phase I and phase II clinical trial including patients with hepatitis C virus infection [133,134]. Since long non-coding RNAs and circular RNAs have been investigated within the last decade, long non-coding RNA- or circular RNA-targeting therapeutics have not entered into clinical trials yet. Although non-coding RNA therapeutics can be a novel approach to treat ROP, there are several challenges in developing non-coding RNA therapeutics. Non-coding RNAs may bind with various targets and initiate undesirable effects. Moreover, RNA therapeutics can be unstable and readily degradable and may not pass cell membrane due to their negative charges. However, this obstacle can be overcome by applying a variety of chemical modification and using delivery method of encapsulated carriers such as liposomes [135,136,137,138].

### 5.2. Cell Therapy

Stem cells, pluripotent cells with self-regenerating potentials, have been investigated in ROP (Figure 2). Various types of stem cells, such as bone marrow (BM)-derived endothelial progenitor cells, BM-derived myeloid progenitor cells, and BM-derived monocyte lineage cells, inhibit RNV in the OIR model. Outgrowth endothelial cells, a subtype of endothelial progenitor cells, play a role in vascular repair. Administering outgrowth endothelial cells to vessels increases normal vessel development and inhibits pathological neovascularization in the mouse ROP model [139]. Moreover, a reduction in the number of endothelial progenitor cells is associated with a disruption in normal retinal vascularization [140]. According to Ritter et al., cell therapy utilizing BM-derived myeloid progenitor cells can be used to treat ROP in a clinical setting. They reported that BM-derived myeloid progenitor cells could move to the region of the retina lacking blood vessels, promoting normal retinal vessel development [141]. In addition, BM-derived monocyte lineage cells can differentiate into endothelial cell-like cells. They can migrate to avascular areas and subsequently facilitate regular intraretinal revascularization and inhibit abnormal blood vessel development [142,143].

To date, stem cell therapies have been investigated in preclinical and clinical studies of various type of ocular diseases such as retinopathy of prematurity, age-related macular degeneration, and diabetic retinopathy [144,145,146]. Administration of endothelial cells originated from human-induced pluripotent stem cells inhibited vessel loss and retinal angiogenesis in OIR mice model [145]. The efficacy and safety of BM-derived mesenchymal stem cells was investigated in a clinical trial including patients with diabetic retinopathy. Compared with the patient group having proliferative diabetic retinopathy, the patient group with non-proliferative diabetic retinopathy had significant improvement in visual acuity [144]. Implantation of embryonic stem cell-derived RPE was found to improve visual acuity in patients with severe neovascular AMD in a phase I clinical trial [146]. Even though stem cell transplantation showed protective and regenerative effects in ocular diseases, adverse effects of intravitreal stem cell injection such as retinal detachment and vision loss were reported [147,148]. Thus, the optimal route of administration of stem cells needs to be tested to deliver stem cells safely. Additionally, long-term effects of stem cell therapy need to be evaluated to consider its use in clinical settings.

### 5.3. Gene Therapy

Gene therapy using viral vectors is a potential therapeutic strategy to treat ROP (Figure 2). Lentiviruses, adenoviruses, or adeno-associated viruses are generally used as viral vectors to transfer genes to the retina. Because of its low immunogenicity, adeno-associated virus is the most commonly used vector for humans [149]. In addition, infection by an adeno-associated virus vector containing an anti-angiogenic gene such as endostatin, pigment epithelium-derived factor, or tissue inhibitor metalloproteinase-3, suppresses RNV in the mouse ROP model [150]. Since the response to viral vectors may vary depending on species, testing their effects on humans is critical. 

Meanwhile, gene editing using clustered regularly interspaced short palindromic repeats (CRISPR)-CRISPR associated protein 9 (Cas9) endonucleases can be another approach to treat retinal angiogenesis. It can alter the target gene at the DNA level and inhibit angiogenesis indefinitely [151]. Various angiogenic genes, including those encoding VEGF-A, VEGFR2, and hypoxia-inducible factor 1 subunit alpha, can be inhibited using the CRISPR-Cas9 system in the in vitro and in vivo models of retinal disease [152,153,154,155,156,157]. Since clinicians may be reluctant to use therapies involving permanent changes in genes, Cas13 has been developed. Cas13, a CRISPR endonuclease targeting RNA, inhibits RNA efficiently with fewer off-target effects [158].

Gene therapy can be an attractive therapeutic option to treat ROP since it can provide long-term pharmacological effects and may not need frequent administration of drugs. Voretigene neparvovec is the first FDA-approved adeno-associated virus gene therapy to treat inherited retinal dystrophy caused by mutation of the RPE65 gene [159]. Currently, numerous gene therapies are in clinical trials for ocular diseases including age-related macular degeneration, diabetic macular edema, glaucoma, and hereditary ocular diseases [160]. For example, sFLT-1, a potent inhibitor of VEGF, containing a recombinant adeno-associated vector was investigated in a phase I clinical trial including patients with neovascular age-related macular degeneration [161]. Although gene therapy may be a promising therapeutic option to treat ROP with long-lasting effects, the optimal dose of gene therapy needs to be investigated to minimize its toxicity and immunogenicity. 

## 6. Conclusions

ROP is a complex progressive retinal disorder involving multiple factors, such as growth factors, hormones, and metalloproteinase. Despite the application of various pharmacologic and non-pharmacologic therapies to ROP, the incidence of ROP has increased. As a result, ROP is one of the major causes of childhood blindness. Although laser photocoagulation is a standard therapy to treat ROP and anti-VEGF drugs are available, these therapies have drawbacks. Laser therapy may cause myopia and unfavorable ocular outcome, whereas anti-VEGF therapies may have long-term systemic effects on other organs. Thus, it is important to identify novel therapeutics for ROP treatment. Recently, non-coding RNAs, cell therapy, and gene therapy have been shown to regulate ROP, indicating their potential application in the disease. Therefore, it is necessary to investigate the precise role of these emerging therapeutics and therapeutic strategies in ROP treatment.

## Figures and Tables

**Table 1:** Anti-VEGF agents used to treat retinopathy of prematurity.

Anti-VEGF Drug	Drug Class	Target Specificity	Mechanism	FDA-Approved Ophthalmic Indications	Molecular Weight	Relevant Study
Bevacizumab	Monoclonal antibody	All VEGF-A isoforms	Binds to VEGF and suppress interaction between VEGF and Flt1 and KDR receptors	None	149 kD	[46,51]
Ranibizumab	Monoclonal antibody fragment	All VEGF-A isoforms	Binds to VEGF-A and inhibit retinal angiogenesis	AMD, DME, RVO, DR, mCNV	48 kD	[47,52,53]
Pegaptanib	RNA aptamer	VEGF-A_165_	Binds to VEGF-A_165_ and inhibit RNV	AMD	50 kD	[54]
Aflibercept	Fusion protein	All VEGF-A isoforms, VEGF-B, and PlGF	Bind to VEGF-A, -B, and PlGF and suppress RNV	AMD, RVO, DME, DR	115 kD	[55,56,57]

AMD, age-related macular degeneration; DME, diabetic macular edema; DR, diabetic retinopathy; Flt1, FMS-like tyrosine kinase 1; KDR, kinase insert domain receptor; mCNV, myopic choroidal neovascularization; PlGF, placental growth factor; RNV, retinal neovascularization; RVO, retinal vein occlusion; VEGF, vascular endothelial growth factor.

**Table 2:** The list of ROP therapies under investigation.

Agents Acting as Inhibitors of ROP	Agents with Contradictory Results in ROP
Beta-blocker	Antioxidants
Caffeine	Corticosteroids
Polyunsaturated fatty acids	Light
Vitamin A	NSAIDs
	Oxygen
	Serum IGF-1

IGF-1, insulin-like growth factor 1; NSAIDs, non-steroidal anti-inflammatory drugs.

**Table 3:** The role of ROP therapies under investigation with partially proven results.

ROP Regulator	Mechanism	Effects on ROP	Major Findings	Reference
Beta-blocker	Downregulate VEGF and IGF-1 by blocking β-adrenoreceptors	Inhibit	-Oral and topical formulations of propranolol inhibit the progression of ROP	[69,70,71,72,73,74]
Caffeine	Suppress VEGF and MMPs	Inhibit	-Oral administration of caffeine reduces the incidence of severe retinopathy -Early caffeine intake reduces the risk of ROP requiring laser therapy	[75,76,77,78]
Polyunsaturated fatty acids	Reduce TNF-α	Inhibit	-Omega-3-polyunsaturated fatty acid supplementation inhibits pathological angiogenesis in OIR mice model -Polyunsaturated fatty acids intake improves visual acuity in infants and lowers the risk of severe ROP	[79,80,81]
Vitamin A	Inhibit VEGF	Inhibit	-Vitamin A inhibits pathological neovascularization in the OIR rat model by reducing VEGF -Vitamin A supplementation reduces the progression and incidence of ROP	[82,83,84,85]

IGF-1, insulin-like growth factor 1; MMP, matrix metalloproteinase; OIR, oxygen-induced retinopathy; ROP, retinopathy of prematurity; TNF-α, tumor necrosis factor; VEGF, vascular endothelial growth factor.

## Abbreviations

Ang	Angiopoietin
BM	Bone marrow
HIF	Hypoxia inducible factor
IGF-1	Insulin-like growth factor 1
MMP	Matrix metalloproteinase
OIR	Oxygen-induced retinopathy
RNV	Retinal neovascularization
ROP	Retinopathy of prematurity
VEGF	Vascular endothelial growth factor
